# Consciousness Science Needs Some Rest: How to Use Resting-State Paradigm to Improve Theories and Measures of Consciousness

**DOI:** 10.3389/fnins.2022.836758

**Published:** 2022-03-29

**Authors:** Marcin Koculak, Michał Wierzchoń

**Affiliations:** ^1^Consciousness Lab, Institute of Psychology, Jagiellonian University, Krakow, Poland; ^2^Centre for Brain Research, Jagiellonian University, Krakow, Poland

**Keywords:** consciousness, resting-state, neuroimaging, neural correlates of consciousness, state, content

## Introduction

The discovery of a brain network that is systematically deactivated during task execution while staying active when participants have no task to perform has instigated a new area of studies (default mode network; Raichle et al., 2001). It also imposed on neuroscience a particular dichotomous view of brain activity seen as the constant transitions between a reflexive mode (captured as the neural activity during a task) and a default mode (reflecting intrinsic brain activity; Callard and Margulies, 2011). The first can be studied with various cognitive tasks. The second is most pronounced when the participants are not receiving any external stimulation and have no straightforward task. This so-called “resting-state” (RS) paradigm became the most widely used method to capture the intrinsic, default activity of the brain. It is often treated as a baseline condition (Gusnard and Raichle, 2001) with which the task activity can be contrasted, revealing neural activity related to particular cognitive tasks. Over the years, the notion of the opposition of intrinsic and task activities (Cole et al., 2014) and the concept of baseline activity (Morcom and Fletcher, 2007) received a significant amount of criticism. However, the RS is still used throughout neuroscience, in a mostly unchanged form since its introduction. Recently, some researchers (Finn, 2021) argued that the way we collect RS data should be improved. It was also argued that they should be combined with a task to reflect the more nuanced and complex nature of brain activity.

Here, we would like to discuss the importance of those recent works for the neuroscience of consciousness. Similar to other subfields, the above-mentioned dichotomous view of brain activity is also present in many studies on consciousness. The RS paradigm is primarily used in studies investigating the states of consciousness. It is treated as a baseline condition for comparing the activity in, for example, the disorders of consciousness (Hannawi et al., 2015), sleep (Tagliazucchi and Laufs, 2014), or anesthesia (Boveroux et al., 2010). This approach is consistent with the view treating RS activity as a measure of wakefulness (or general excitability of the brain). This view drastically limits RS usefulness, seeing it as a minimal but insufficient prerequisite for conscious processing. Here, we argue that the new reformative methodological approach (Finn, 2021) can be conceptually applied to consciousness research so as to allow for a better understanding of its neuronal underpinnings, especially in the emerging field investigating the relations between its states and contents (Bachmann and Hudetz, 2014). We also propose how such an improved RS paradigm should look like and how it may benefit the scientific study of consciousness.

## Consciousness at Rest

The RS already proved itself useful in consciousness research, for example, improving the diagnostics of various disorders of consciousness (Bai et al., 2017). This success stems directly from the design of the RS paradigm. At the minimal level, it only requires some neuroimaging apparatuses as no stimulation is presented to the participant. It can be administered for long periods of time without much fatigue, allowing for a more thorough assessment (which can be crucial for diagnosing, e.g., epileptic seizures). Finally. it does not require any involvement from the participant who, depending on the situation, might not be able to properly perceive stimulation or provide a response. This restrictive approach minimizes the complexity of the procedure, yielding more robust results and making it more convenient in clinical settings.

This method is, however, usually transferred directly into non-clinical settings, where there is little practical necessity for such minimal procedures, except the convenience for researchers. While it might not have much consequence when investigating, for example, the connectivity of particular brain regions, in the case of consciousness research, it can very well-obstruct more than it is revealing. Firstly, we should consider what aspect of consciousness we can capture this way. In a clinical setting, the focus is on distinguishing between the states of consciousness and unconsciousness. Assuming that this disposition is relatively stable in time, researchers test a variety of characteristics through the calculation of single-digit outcomes averaged across the whole recording (Sitt et al., 2014). These outcomes are then used to classify patients as conscious or unconscious. However, when testing healthy awake participants, the variability in the signal cannot reflect a wakefulness or consciousness state but arises primarily from the conscious content present in their minds. Even if the RS session is short, this content will fluctuate significantly during this time, especially since no explicit task is given. Therefore, running a minimal RS procedure and averaging the results over the whole sessions blur content-related correlates and exaggerates unrelated to the consciousness differences between the participants. From this perspective, it seems crucial for consciousness research to stop ignoring this subjective variability. It appears that the easiest way to start is to ask the participants about it (Gonzalez-Castillo et al., 2021). This simple idea can result in many useful approaches, some of which we will point out in the next section.

The second troubling assumption implicitly included in the classical RS approach is that an acquired signal represents a typical spontaneous activity. However, the whole situation of a scientific experiment is undoubtedly very far from everyday life. Participants are usually confined in a small space of an experimental booth or MRI machine with dimmed lights and an explicit instruction to “not think about anything in particular.” This is a very artificial setting compared to everyday life. For a long time, we knew of such specific influences (e.g., experimenter effect; Rosenthal, 1976) and it seems reasonable to assume that this effect influences brain activity in the same way as behavior. This could not only alter the pattern of spontaneous thought that the participants usually experience (e.g., focusing on the novelty or oddness of the situation) but also influence the correlates of wakefulness (e.g., increasing the level of stress related to taking part in an experiment). Introducing an RS that more closely resembles everyday life activities could help to verify the generalizability of already established markers and test to what degree they depend on the factors not related to consciousness.

The final troubling implicit assumption is treating rest and task as exclusive modes of operation. An RS procedure is a form of a task just by the virtue of being a part of an experiment. Conversely, assuming that all changes in the signal during a task are related only to how it is constructed significantly limits our inferences. This can be observed in the research on the neural correlates of consciousness, where the trials with weak or absent stimuli are usually labeled as unconscious (Silverstein et al., 2015). However, it is safe to assume that the participants were in fact conscious of many things (the screen, their bodies, etc.), not just the stimulus. Experiments are constructed to keep external conditions maximally constant, but the internal conditions, which most certainly vary, typically go unaccounted for. Extending the definition of rest to all the activities that are not directly influenced by the experimental manipulation, we can imagine how this background activity could behave similarly to an explicit RS procedure. These background-conscious contents are also likely to fluctuate and change, interacting with task-related processes without researcher control. The averaged evoked activity would then present a false image of stable markers that might replicate poorly in different conditions or with different individuals. The inclusion of the non-task signal into the analysis could at least account for those changes.

## Making Rest More Useful

We believe that analyzing an RS activity is a necessary element to capture the phenomenon of consciousness in its entirety. Currently, however, resting procedures are mostly done with the minimal clinical approach mentioned earlier. Therefore, unlocking its full potential will not only require to introduce more complexity to its design, which will inevitably make the analyses more difficult, but it will also allow for more informed inferences about the underlying mechanisms.

The most straightforward improvement is the inclusion of some form of experience sampling (Martinon et al., 2019). By simply asking the participants what they did during the resting period, we could account for the different thought patterns that a participant might have when being prompted to wait and “do nothing.” Inevitably, people will vary greatly in the content of their thoughts. Depending on the form of the RS procedure and goal of the researchers, there are different ways that this could be done. When the main focus of an experiment are the spontaneous thoughts and their dynamics, an occasional prompt asking for the content of thought might be a viable option (e.g., Hurlburt and Akhter, 2006). When only a general type of the thoughts during resting is of interest, a questionnaire (e.g., Amsterdam Resting State Questionnaire; Diaz et al., 2014) at the end might give the researchers enough information. In fact, self-reports seem to be reliably related to the activity of major brain networks (Stoffers et al., 2015). Most importantly, this augmentation allows for tracking the correlates of spontaneous conscious thought on top of the markers of excitability.

Another enhancement is a more ecological approach to RS sessions. Instead of maximally reducing the external stimulation, researchers can incorporate settings resembling the natural human environment (Sonkusare et al., 2019). This can be achieved by the passive experiencing of audiovisual material (Naci et al., 2014), usage of more immersive technologies like virtual reality (Baumgartner et al., 2006), or recording resting data outside of the laboratory (Edwards and Trujillo, 2021). Not only would it bring the brain's activity closer to what is actually going on most of the day; it would also impose some level of constraint on the spontaneous thought. Humans evolved to function in a certain environment, the elements of which naturally capture attention and invoke certain cognitive processes. From this perspective, unconstrained spontaneous thought is also not something that happens often. Therefore, by introducing stimulation to a session, we can measure a structured version of RS, where the order of cognitive processes is dictated from the outside (Hasson et al., 2004), rather than reconstructed through experience sampling. Some researchers already tried this, for example, by *post-hoc* analyzing task data in a continuous fashion (Sitt et al., 2014), but most of its potential is yet to be explored.

Finally, following the aforementioned principles, one could introduce paradigms combining the RS with a task. Such integrated designs would allow us to investigate the relation between the conscious task-related content with the state. There is already a substantive literature on how the pre-stimulus activity influences conscious perception (e.g., Benwell et al., 2017; Samaha et al., 2017). Similarly, we could also look for stimulus-related patterns that reverberate in a non-task activity after its presentation. For example, through introducing randomly short breaks between stimuli, we could investigate if and how stimulation influences the background activity or even sample experience to get reports on fatigue, engagement, and other potentially important factors (Thompson et al., 2013). On top of that, as acknowledged by Finn (2021), in principle, there is no reason to not analyze task data with methods devised for the RS. Analyzing task data using RS methods might be more demanding than evoked activity analyses, but it could bring an important insight into conscious brain activity that is being overlooked.

## Challenges

The main methodological challenge we see is the necessity to rework both experimental paradigms and analysis pipelines. This might be difficult since most studies rely on evoked activity and model only the stimulus-related conscious activity of the brain. As mentioned in the previous section, we think that employing a mixed resting-task approach could be a good first step to recognize the most promising aspects of the data, for which new more sophisticated paradigms and analyses methods could be devised.

We also acknowledge that currently, there are significant theoretical obstacles for incorporating RS analyses. Most of the prominent theories of consciousness were constructed and validated through experiments involving tasks and stimuli-evoked activity. Therefore, it is difficult to formulate any testable hypotheses based on them, making the use of RS procedures troublesome. It seems that for now, only few (e.g., Tononi et al., 2016; Bachmann et al., 2020) could provide any predictions. Yet, we are convinced that this not only poses a challenge for current theories but also gives an opportunity for broadening their scope to more fully describe consciousness and the mechanisms that govern it.

## Conclusions

In this opinion paper, we advocated for the greater use of the RS paradigm in consciousness research. We believe that this will improve the existing models of and analytical approaches to consciousness, allowing to better understand its neural mechanisms. It is also a necessary step to account for all aspects of conscious experience that people have during their everyday life.

## Author Contributions

MK drafted the initial version. All authors revised the manuscript. All authors contributed to the article and approved the submitted version.

## Funding

This work was supported by National Science Center Poland (Grants No. 2016/23/N/HS6/00844) awarded to MK.

## Conflict of Interest

The authors declare that the research was conducted in the absence of any commercial or financial relationships that could be construed as a potential conflict of interest.

## Publisher's Note

All claims expressed in this article are solely those of the authors and do not necessarily represent those of their affiliated organizations, or those of the publisher, the editors and the reviewers. Any product that may be evaluated in this article, or claim that may be made by its manufacturer, is not guaranteed or endorsed by the publisher.
